# QTLs for Woolly Poplar Aphid (*Phloeomyzus passerinii* L.) Resistance Detected in an Inter-Specific *Populus deltoides* x *P*. *nigra* Mapping Population

**DOI:** 10.1371/journal.pone.0152569

**Published:** 2016-03-29

**Authors:** Giorgia Carletti, Andrea Carra, Gianni Allegro, Lorenzo Vietto, Francesca Desiderio, Paolo Bagnaresi, Alberto Gianinetti, Luigi Cattivelli, Giampiero Valè, Giuseppe Nervo

**Affiliations:** 1 Council for Agricultural Research and Economics (CREA)-Research Unit for Intensive Wood Production, Casale Monferrato (AL), Italy; 2 Council for Agricultural Research and Economics (CREA)-Genomics Research Centre, Fiorenzuola d'Arda (PC), Italy; 3 Council for Agricultural Research and Economics (CREA)-Rice Research Unit, Vercelli, Italy; CIRAD, FRANCE

## Abstract

The genus *Populus* represents one of the most economically important groups of forest trees. It is composed by approximately 30 species used for wood and non-wood products, phytoremediation and biomass. Poplar is subjected to several biological and environmental threats although, compared to annual crops, we know far less about the genetic bases of biotic stress resistance. Woolly poplar aphid (*Phloeomyzus passerinii*) is considered a main pest of cultivated poplars in European and American countries. In this work we present two high density linkage maps in poplar obtained by a genotyping by sequencing (GBS) approach and the identification of QTLs involved in *Ph*. *passerinii* resistance. A total of 5,667 polymorphic markers (5,606 SNPs and 61 SSRs) identified on expressed sequences have been used to genotype 131 plants of an F1 population *P ×canadensis* obtained by an interspecific mate between *Populus deltoides* (resistant to woolly poplar aphid) and *Populus nigra* (susceptible to woolly poplar aphid). The two linkage maps, obtained following the two-way pseudo-testcross mapping strategy, have been used to investigate the genetic bases of woolly poplar aphid resistance. One major QTL and two QTLs with minor effects (mapped on LGV, LGXVI and LG XIX) explaining the 65.8% of the genetic variance observed in the progeny in response to *Ph*. *passerinii* attack were found. The high density coverage of functional markers allowed the identification of three genes belonging to disease resistance pathway as putative candidates for *P*. *deltoides* resistance to woolly poplar aphid. This work is the first report on genetic of woolly poplar aphid genetic resistance and the resistant loci associated markers identified represent a valuable tool in resistance poplar breeding programs.

## Introduction

The genus *Populus* (poplars, cottonwoods and aspens, collectively known as poplars) is composed by approximately 30 species widely distributed in the Northern Hemisphere. Poplar is grown for wood products (timber and paper pulp production, biomass), and for phytoremediation purposes, representing one of the most economically important group of forest trees [1]. *Populus* has been chosen as the model genus for forest plants in genetic and genomic studies, owing to its small genome size (~500 Mb organized in 19 chromosomes *vs*. 15–35 Gb of most conifers and others forest tree species), a physical/genetic distance ratio of roughly 220 kb/cM (essentially the same as *Arabidopsis*, [2]), rapid growth and seed development and easy vegetative propagation. Many genetic and genomic resources are available such as the genome sequence of *Populus trichocarpa* (http://www.plantgdb.org/PtGDB [3]), linkage maps [2,4–6] and microarray-based genotyping platforms [7].

Poplar is subjected to several biotic and environmental threats affecting the growth and development of the plants and consequently the quantity and quality of useful yield. Among them, pathogens (e.g. leaf rust, leaf spot) and pests, above all the woolly poplar aphid (wpa), *Phloeomyzus passerinii* (Signoret, 1875), have a main impact on yield and wood quality estimated to cause up to 10% reduction of potential production. Wpa is very harmful to poplar plantations in several European and American countries where the majority of cultivated clones are susceptible [8]. Typically, wpa starts the colonization in bark crevices of old poplar plants, often at the insertion of the lowest branches 5–6 meters above the ground. Aphid colonies are revealed by abundant waxy flocks, woolly and whitish in colour, which are produced mainly by apterous females. Feeding activity, which involves the injection of a toxic saliva, causes bark suberization and, during the heaviest attacks, cracks and necrosis. The death of the bark causes the interruption of water and nutrients circulation, determining progressive damages [9]. Little is known about the feeding physiology of wpa and about the genetic variability of the pathogen populations [10–11]. Similarly, the mechanism and genetic bases of the host plant resistance to this pest are still poorly known, although many studies have been devoted to the description of the resistance behavior of cultivated poplar clones [12]. *Populus* spp. exhibits different levels of resistance to wpa, with high levels of resistance observed in *P*. *deltoides* [13] and varying levels of susceptibility observed in *P*. *nigra* [10].

Currently, a number of genetic maps are available in poplar although no loci controlling wpa resistance have been described. The first map, developed by Bradshaw *et al*. [2], containing a total of 343 markers (215 RFLPs, 17 STSs, 111 RAPDs) covered the 50% of the genome. After the publication of the poplar complete genome sequence [3], a growing set of SSR markers were made available allowing the construction of dense linkage maps [14–15]. Cervera *et al*. [4] reported a dense genetic map for *P*. *deltoides*, *P*. *trichocarpa* and *P*. *nigra*, based on few hundred AFLPs and about one hundred SSRs. Yin *et al*. [16] expanded the genome coverage developing a linkage map in *P*. *deltoides* × *P*. *euramericana* F1 population using 839 markers (AFLPs, RAPDs and ISSRs).

The detection of markers tightly associated to specific traits is more efficient in molecular maps developed from large populations coupled with marker technologies allowing map saturation. With the diffusion of Next Generation Sequencing technologies, high throughput genotyping with thousands of markers has become relatively easy, rapid and cost effective [17–18]. For instance, the identification of thousands of Single Nucleotide Polymorphisms (SNPs) has increased speed and accuracy of QTL mapping in a very cost effective way [19–20].

In this work we present the development of two high density linkage maps in poplar obtained through a RAPiD-seq genotyping procedure [21]. The linkage maps have then been used to investigate the genetic bases of wpa resistance, identifying three major QTLs and underlying putative candidate genes.

## Material and Methods

### Aphids, plant material and DNA extraction

Aphids were taken from a laboratory colony, established from wild apterous parthenogenetic colonies collected from CREA-Research Unit for Intensive Wood Production, Casale Monferrato (Italy) farm (geographical coordinates, longitude 8.50538/latitude 45.13300) on *Populus* x *euroamericana* "I-214" genotype. No permission was required for sampling in the areas where the aphids were collected. The colony was maintained under controlled conditions according to protocol on stem cuttings of the genotype I-214 [10].

A mapping population represented by 131 hybrid poplars *P* ×*canadensis* was generated by controlled mating of *P*. *deltoides* D0-092b (female and wpa resistant parent) x *P*. *nigra* N074 (male and wpa susceptible parent). *P*. *deltoides* species is native of the Nearctic ecozone, *P*. *deltoides* D0-092b was imported from Oklahoma State, USA, and selected for growth and wpa resistance traits in Italy. *P*. *nigra* species, instead, has a Palaearctic distribution and the parental line N074 was collected in Val di Susa, Turin, Italy and was selected for rust resistance and sylleptic branchiness in Italian breeding programs. Plants were grown and maintained in open field at the CREA—Research Unit for Intensive Wood Production, Casale Monferrato (AL), Italy. Total genomic DNA was isolated from young leaves using GenElute^™^ Plant Genomic DNA Miniprep Kit (Sigma Aldrich) according to the manifactures’ instructions. DNA concentrations were determined using Qubit Fluorimeter (Invitrogen) and DNA quality was checked on 1% agarose gel.

### Microsatellite (SSRs) genotyping

130 SSRs of *P*. *trichocarpa* uniformly distributed on whole genome were selected from previous works [5, 22–24]. A preliminary screening of the selected SSRs was carried out on the two parental lines and six individuals of progeny to verify the polymorphism between *P*. *nigra* and *P*. *deltoides* and test the segregation in the progeny. A total number of 47 tested SSRs was discarded after these evaluations.

SSR amplifications were performed as described by Schuelke [25] with minor modifications. The PCR amplifications were carried out in 10 μl volumes containing 10 ng genomic DNA, 1× PCR buffer, 1.5 mM MgCl _2_, 0.4 mM dNTP mix, 0.25 μM forward primer including a 5' M13 tail, 2.5 μM reverse primer, 1 μM of 5'[FAM] or 5'[HEX]-labeled M13, 1U Dream Taq DNA polymerase (Thermo Fisher Scientific). A unique thermo-cycling protocol was used for all SSR primers: initial denaturation at 94°C, 3 min; 6 cycles of touch-down PCR including: 94°C, 30s; 56/53.5°C, 30s (-0.5°C per cycle); 72°C, 45s, followed by 33 cycles including: 94°C, 30s; 53°C, 30s; 72°C, 45s, with a final extension at 72°C for 10 min. The M13-tailed primer method [26] was used to label amplicons for the visualization of PCR products on capillary DNA analyzer. Forward primers used were 5’-tailed with 19-base pair M13 sequence (5’-CACGACGTTGTAAAACGAC-3’), tagged in 5’ end with 6-carboxyfluorescein (6-FAM) or hexachloro-carboxyfluorescein (HEX). The microsatellites were multiplexed and analyzed on GA3500 (Applied Biosystem) automated capillary electrophoresis instrument using GeneScan ROX 500 as size standard. Visualizations and sizing of the PCR fragments were performed using the GeneMapper software version 4.1 (Applied Biosystem).

### SNP genotyping

SNPs genotyping was performed by RAPiD Genomics (http://www.rapid-genomics.com) using the Randomly Amplified Polymorphic DNA Sequencing (RAPiD-seq) GBS protocol. Random amplifications of genomic DNA, sequencing and SNP calling were run by RAPiD Genomics following internal protocols [21]. The polymorphic SNPs were localized on the *Populus trichocarpa* genome scaffolds (genome sequence available, [3]) and named by two numbers separated by an underscore. The first number represents the number of scaffold conferred by RAPiD-seq genotyping, while the second one is a progressive number identifying the physical position in bp of each SNP on the scaffold.

### Marker score, segregation analysis and map construction

Linkage analysis was developed using SNP markers showing a segregation ratios of 1:1 (test cross segregation) or a segregation ratio of 1:2:1 (F2 ratio), as well as SSRs following both a segregation 1:1:1:1 (full cross segregation) or 1:1. Markers with more than 10% of missing data were discarded and not used for map development. A total of 73 SSR and 5,698 SNP polymorphic markers were used for map construction following the two-way pseudo-testcross mapping strategy [27], running JoinMap 4.0 software [28]. A full-sib family from a cross between two heterozygous parents was used as population type. The two linkage maps, one for each parental genotype, were independently calculated following a two steps procedure. Segregation distortion for all markers was assessed using a χ^2^ analysis. Markers were considered distorted when a significant (P>0.05) deviation from expected Mendelian segregation was observed, but they were included in the linkage analysis if their presence did not alter surrounding marker order in a given linkage group.

During the first step, the linkage groups were identified using the following parameters: LOD threshold ≥ 8.0, recombination fraction threshold of 0.30, ripple value of 1.0 and jump threshold of 5.0. The LOD score was selected based on the number of groups formed for each LOD value. Kosambi's mapping function and Maximum Likelihood (ML) algorithm were used for map construction. In the second step, each linkage group was analyzed with an intra-group analysis using LOD threshold ≥ 3, recombination fraction threshold of 0.35, ripple value of 1.0 and jump threshold of 5.0. A restricted number of markers resulting unmapped in the first step (12 SNPs segregating 1:2:1 and 6 SSRs segregating 1:1) were reinserted one by one adding them to their respective linkage group, on the bases of their physical position identified through GBS protocol. The resulting linkage maps were drawn using the MapChart 2.2 software [29].

### Aphid resistance test and data statistical analysis

**Fig 1:**
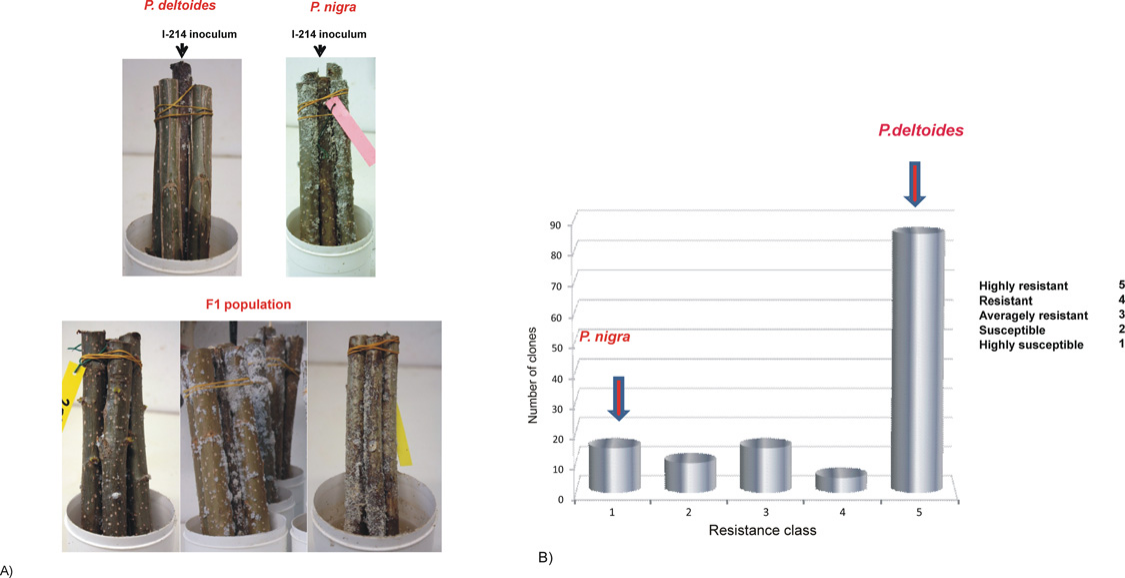
*Ph*. *passerinii* infection on *P*. *nigra*, *P*. *deltoides* and derived segregation population. (A) Aphid resistance test on the two parental lines (upper panel) and on some individuals of F_1_ population belonging to infection classes 4, 2, 1 (lower panel, respectively from left to right). (B) Frequency distribution of the phenotypic classes in *P* ×*canadensis* F_1_ population. The five resistance classes are plotted on the horizontal axis, while the vertical axis reports the number of clones in each class.

133 genotypes (*P*. *deltoides* female parent and *P*. *nigra* male parent, and 131 progeny plants *P* ×*canadensis*) were subjected to a laboratory bio-assay to test their resistance to wpa according to established procedures [10]. The artificial inoculation assay was based on 8 biological replications (cuttings) for each genotype, divided in 2 groups of 4, evaluating both the surviving insects and the progeny produced 28 days after inoculation. As shown in Fig 1 each group was composed by one cutting of poplar clone ‘I-214’ surrounded by four cuttings of the testing genotype. The cutting clone ‘I-214’, highly susceptible to wpa, was inoculated and used as a spreader in each group. During the bio-assay, cuttings were kept in an isolated laboratory box under plastic jars with bottom immersed in water, using controlled conditions (temperature 20–22°C and relative humidity of about 70%) and natural day length conditions (month March, light 11 hours and dark 13 hours). The intensity of biological response was evaluated by visual check, attributing to each cutting a score on 0 to 4 scale. Each value of the scale was obtained calculating the log5 of the inferior value of each class and then it was arranged in an evaluation scoring, ranging from 1 (highly susceptible) to 5 (highly resistant), Table 1. For each genotype, the level of aphid infestation was estimated by the size of the leaf surface colonized by the insects. The mean infestation value of each genotype was normalized on the mean value of the spreader clone ‘I-214’. This normalization procedure was applied because the high number of cuttings did not allow a simultaneous screen of all samples, whose infestation values changed according to the evaluation time.

**Table 1:** The infection class in woolly poplar aphid laboratory assay.

n° of aphids estimated (Class)	Infestation value (log_5_ of the lower class limit)	Genotype mean / ‘I-214’ mean	Score value	Evaluation
<5	0*	0.00–0.20	5	Highly resistant
5–24	1	0.21–0.40	4	Resistant
25–124	2	0.41–0.60	3	Averagely resistant
125–624	3	0.61–0.80	2	Susceptible
>624	4	>0.80	1	Highly susceptible

*assigned also to cuttings with no aphids

Statistical analysis was performed by using GenStat 17 software. The normality of the distribution of phenotypic data was assessed by Shapiro-Wilk test and a Kruskal Wallis statistical test was performed to determine if differences of infection values between genotypes and blocks were statistically significant, considering the 2 groups of genotypes as two different blocks.

### QTL mapping

QTL analysis was performed on *P*. *nigra* and *P*. *deltoides* data separately using the R/qtl module of the R statistical package [30]. A genome-wide significance level of 5% was used and a LOD threshold was determined after 1000 permutations. The search for putative candidate genes was then focused on the chromosome regions of the QTLs, investigating a genetic interval defined by a LOD greater than the maximum LOD minus 1, called as the LOD-1 confidence interval region.

### Bioinformatics analysis and candidate gene investigation

The R Biostrings (Bioconductor) package was used to import genomic sequences and facilitate sequence and genomic data handling. In order to assess SNP localization in genes, a DNAStringSet instance (Biostrings package) of Ptrichocarpa_156_gene.gff3.bz2 (v.2, http://www.plantgdb.org/XGDB/phplib/download.php?GDB=Pt) was created. Subsequently, a further DNAStringSet was built by filtering for relevant SNP scaffold and coordinate data. This further set contained, for each SNP, 1000 bp of flanking sequences (500 bp upstream and downstream of SNP). A fasta file was generated and used as query for Blast searches against Poplar cDNA (Populus_trichocarpa.JGI2.0.26.cdna). Blast was conducted at various Expect values (1e-30, 1e-70, 1e-100) and gap open penalty, nucleotide mismatch penalty, gap extension penalty were all set to -10.

SNP maps, diagrams of SNP localizations and further graphics were obtained with R custom scripts (R version 3.02) implementing R default graphic functions.

For gene annotation we referred to PtDB v.2 Loci/Annotations (http://www.plantgdb.org) file.

## Results

### Response to woolly poplar aphid infection

High levels of resistance (class 5) were observed in *P*. *deltoides*, where no aphid were able to colonize the trunk in laboratory bio-assay (controlled environment) and in open field. On the contrary, *P*. *nigra* was highly susceptible (class 1, [10]).

All 131 *P* ×*canadensis* F1 plants were infected with wpa using an inoculum derived from the susceptible spreader I-214 clone and their response was analyzed 28 days after the infection. Frequency distribution of the phenotypic classes indicated that no living aphids were observed on 65.6% of *P* ×*canadensis* population (phenotypic class n. 5), highlighting that the major part of the segregating population was highly resistant to wpa. Only the 11.4% *P* ×*canadensis* genotypes belonged to class 1, resulting highly susceptible. The remaining plants, characterized by intermediate levels of resistance/susceptibility, were included in class 2 (7.6%), class 3 (11.4%) and class 4 (3.8%) (Fig 1). A non normal distribution of phenotypic data was observed which was confirmed by a Shapiro–Wilk test (W = 0.7091, P <0.001).

Two Kruskal-Wallis one-way analyses of variance were then performed, which showed a significant variation among genotypes (P<0.001) indicating that the F_1_ individuals had a different response to wpa infection. Instead, no statistical differences were revealed between blocks (P = 0.906) (Table 2).

**Table 2:** Kruskal-Wallis analysis of variance in the mapping population. The variance between blocks shows no significant values.

	Mean Rank	df	P-value
Genotype	923.776	130	<0.001
Block	126837.500	1	0.906

### Markers analysis and map construction

A total of 7,645 SNPs segregating 1:1, 37 SNPs segregating 1:2:1 and 83 SSRs segregating mostly 1:1 and in minor proportion 1:1:1:1 were used to genotype the 131 F_1_ *P* ×*canadensis*. All markers with a distorted pattern having a minor allele frequency (MAF) lower than 0.40, those with more than 10% of missing data and those that altered the surrounding marker order in a given linkage group were discarded before the analysis. Finally, a total of 5,606 SNPs and 61 SSRs were used to build the high-density linkage maps, following the two-way pseudo-testcross mapping strategy [27], leading to the development of two genetic maps, one for each parent. All markers segregating 1:1 were genotyped only on one parent line, while those segregating 1:1:1:1 and 1:2:1 were common to *P*. *nigra* and *P*. *deltoides* and therefore were used to develop both maps (Table 3). A total number of 2,847 markers were mapped on *P*. *nigra* and 2,848 on *P*. *deltoides* maps (Table 4).

**Table 3:** Molecular markers assessed in this work.

Molecular markers selected for mapping	SSR markers	%	SNP markers	%
Number of markers	130		7645	
Monomorphics	47	36.1	/	
Polymorphics	83	63.9	7645	100
1:1 segregation	65	78.3	7608	99.5
1:1:1:1 segregation	18	21.7	/	
1:2:1 segregation	/		37	0.5
Eliminated after MAF and MD values	10	26.5	1947	25.5
Markers used for map construction	73		5698	

MAF, minor allele frequency; MD, mendelian distortion

**Table 4:** A summary of molecular markers reported in the two linkage maps.

Molecular markers included in the two linkage maps	*P*. *nigra*	*P*. *deltoides*
Unlinked (SNPs + SSRs)	30	49
1:1 segregation (SNPs + SSRs)	2847	2867
1:1:1:1 segregation (SSRs)	18	18
1:2:1 segregation (SNPs)	12	12
Markers mapped on parental genomes	2847 (2817 SNPs + 30 SSRs)	2848 (2799 SNPs + 49 SSRs)

In both parents, markers were grouped into 20 Linkage Groups (LGs), as reported in S1 Fig. In both maps, LGI (the longest LG in the poplar genome, [3]) was divided in two groups (LGI-1 and LGI-2). Markers on *P*. *deltoides* map were distributed on 2,745.2 cM with a genome-wide mean inter-locus separation of 2.3 cM, whereas markers on *P*. *nigra* map were distributed on 3,004.9 cM with an average density of one marker per 2.4 cM. The largest gaps were localized in the LGXVI of *P*. *nigra* (25.3cM) and in the LGX of *P*. *deltoides* (19.1 cM). The number of markers, their density and the mean of markers mapped on each LG of two genetic maps were almost the same, nevertheless, the total map length of *P*. *nigra* was 259.7 cM larger compared to the *P*. *deltoides* one. The means marker loci per LG were 142 for both maps while the mean of chromosome map length was 137.3 cM (*P*. *deltoides*) and 150.2 cM (*P*. *nigra*). The largest LG in *P*. *deltoides* map was LGV with 220 markers and a length of 194.8 cM, while in *P*. *nigra* map was LGXVII with 231 markers and a length of 124.2 cM. The smallest LGs in *P*. *deltoides* and *P*. *nigra* were LGXV with 103 markers and 116.6 cM and LGVII with 65 markers and 98.9 cM, respectively.

Segregation distortion of individual markers was observed at 264 loci on *P*. *deltoides* (9.3%) and at 253 loci in *P*. *nigra* (8.9%), as reported in S1 Fig. In several LGs, except in LGVIII, LGXII and LGXIX in *P*. *deltoides* and LGIV, VIII, XI, XVI, XVII, LGXVIII in *P*. *nigra* the deviation from the expected Mendelian segregation was observed. Loci showing segregation distortion in *P*. *nigra* were mostly concentrated in two LGs, LGI-2 (22.5% of the total) and LGIII (28.7% of the total), while in *P*. *deltoides*, distorted markers were mostly observed on LGVI (20.9%), LGX (24.1%), LGXVII (24.4%) and LGXVIII (24.8%). The high values here observed for *P*. *deltoides* LGXVII were in agreement with data previously provided for *P*. *trichocarpa* [5]. Conversely, the high values of segregation distortion on LGs IV and XIX on *P*. *trichocarpa* were not observed in *P*. *deltoides*. The main features of the 20 LGs of both maps are summarized in Table 5.

**Table 5:** Main features of the molecular maps developed in *P* ×*canadensis* F_1_ population.

Linkage Groups	Total n. of markers (n° no-cosegregating markers)		Map Length (cM)		Markers Density (cM/marker)		Segregation distortion markers					
	*P*. *deltoides*	*P*. *nigra*	*P*. *deltoides*	*P*. *nigra*	*P*. *deltoides*	*P*. *nigra*	*P*. *deltoides*			*P*. *nigra*		
	* *	* *	* *	* *	* *	* *	number	%	Favored Allele	number	%	Favored allele
**I-1**	138 (68)	196 (103)	116.2	211.9	1.7	2.1	2	1.4	N	9	4.6	N
**I-2**	134 (56)	89 (40)	100.3	117.4	1.8	2.9	21	15.7	N	20	22.5	D
**II**	214 (112)	193 (103)	215.1	203.6	1.9	2.0	8	3.7	D	42	/	N
**III**	174 (76)	178 (83)	154.5	168.2	2.0	2.0	10	5.7	D	51	28.7	D
**IV**	206 (56)	175 (66)	130.4	164.6	2.3	2.5	2	1.0	N	/	/	
**V**	220 (93)	193 (101)	194.8	222.5	2.1	2.2	29	13.2	N	19	9.8	D
**VI**	158 (83)	214 (88)	198.1	225.9	2.4	2.6	38	24.1		18	8.4	D
**VII**	118 (56)	65 (34)	132.6	98.9	2.4	2.9	12	10.2	D	13	20.0	N
**VIII**	159 (78)	139 (101)	141	183.4	1.8	1.8	/	/		/	/	
**IX**	121 (68)	106 (49)	124.9	108.7	1.8	2.2	3	2.5	D	10	9.4	N
**X**	116 (49)	146 (67)	123.6	166.6	2.5	2.5	28	24.1	D	13	8.9	N
**XI**	116 (49)	133 (59)	136.3	137.7	2.8	2.3	6	5.2	D	/	/	
**XII**	105 (51)	94 (50)	125.7	114.6	2.5	2.3	/	/		16	17.0	N
**XIII**	112 (44)	112 (47)	128.9	116.3	2.9	2.5	15	13.4	N	20	17.9	N
**XIV**	118 (49)	136 (69)	111.6	167.3	2.3	2.4	16	13.6	D	8	5.9	D
**XV**	103 (62)	72 (31)	116.6	93.2	1.9	3.0	3	2.9	D	7	9.7	D
**XVI**	125 (45)	120 (56)	104.2	161.8	2.3	2.9	8	6.4	D	/	/	
**XVII**	135 (44)	231 (75)	109.2	124.2	2.5	1.7	33	24.4	D	/	/	
**XVIII**	125 (47)	124 (35)	140.2	110.8	3.0	3.2	30	24.0	N	/	/	
**XIX**	151 (57)	131 (55)	141	107.3	2.5	2.0	/	/		7	5.3	D
**mean**	142.4	142.4	137.3	150.2	2.3	2.4	16			18		
**Total**	**2,848**	**2,847**	**2,745.2**	**3,004.9**			**264**			**253**		

N, *P*. *nigra allele*; D, *P*. *deltoides allele*

**Fig 2:**
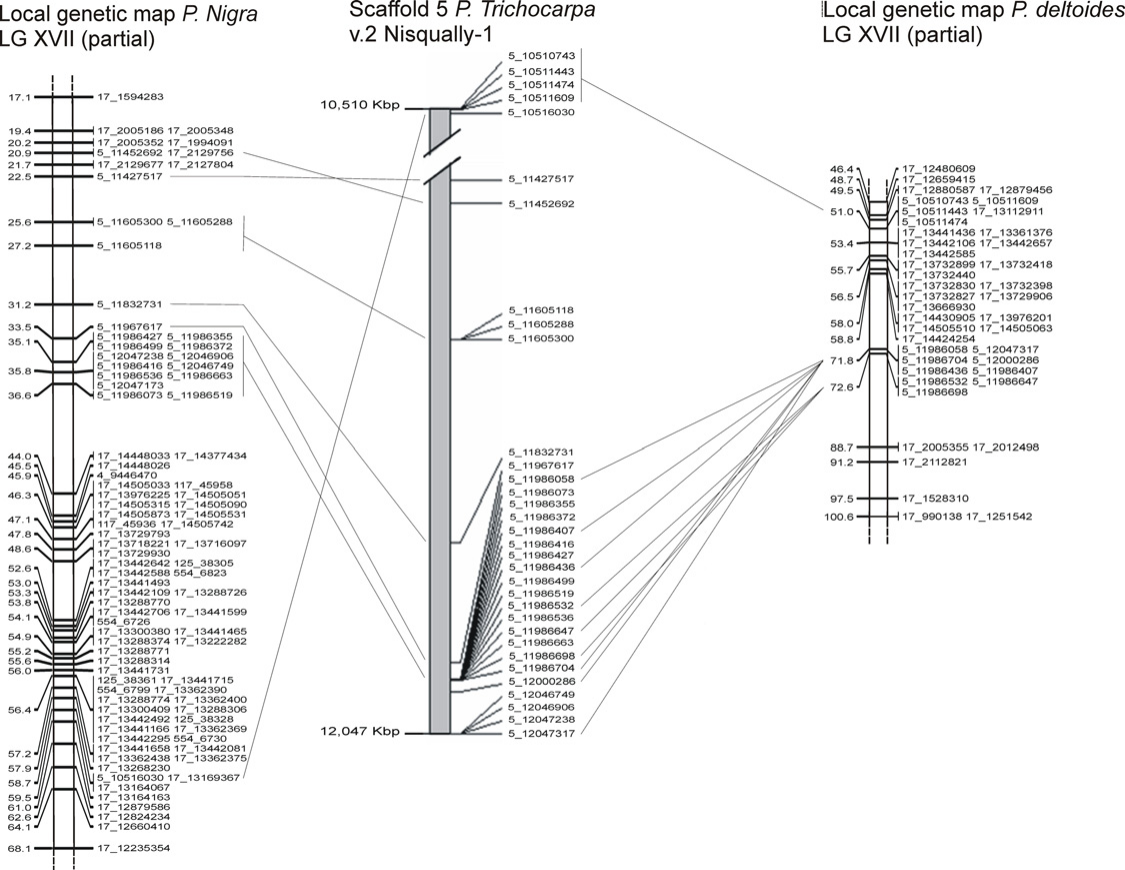
Local genetic maps of the target region of *P*. *deltoides* and *P*. *nigra* and their alignment onto the scaffold 5 of *P*. *trichocarpa* v2.0. Alignment of SNPs with physical position on scaffold 5 of the *P*. *trichocarpa* genome with their position in the P. *deltoides* and *P*. *nigra* maps. Map distances in cM (Kosambi distances) are indicated on the left of the linkage groups (LGs). On the right of the LG are reported the SNP names, composed by a first number representing the number of scaffold conferred by RAPiD-seq genotyping and a second number identifying the physical position in bp of each SNP on the scaffold.

The alignment of the maps to *Populus* genome sequence, has revealed several rearrangements (duplications or translocations). The level of colinearity between *P*. *deltoides* and *P*. *nigra* maps was investigated, identifying regions of non collinear markers: twenty-six translocated markers on five LGs (LG IV, VI, VII, XVII, XIX) in *P*. *deltoides* and 27 on three LGs (LG VI, XVII, XIX) in *P*. *nigra*. The major rearranged region in both maps was localized on LG XVII. Twenty-one SNPs of *P*. *nigra* and 13 SNPs of *P*. *deltoides* belonging to LGXVII were instead located on *P*. *trichocarpa* chromosome 5. They clustered between 20.9 and 36.6 cM and between 71.8 and 72.6 cM on *P*. *nigra* and *P*. *deltoides* map, respectively (Fig 2).

### Sequence annotation

The position of all SNPs mapped in the two parental maps was investigated to check their localization into putative genes. In S1 Table the list of the annotated genes identified by the SNPs is reported. A bioinformatics analysis was performed blasting the flanking regions of the 5,606 SNPs against *P*. *trichocarpa* cDNA v.2, revealing that almost all SNPs (5,593) were localized into predicted genes. In several cases, the distance among adjacent SNPs was very low (less than 100bp) and few markers were localized in the same predicted gene. Once eliminating the redundant SNPs (e.g. the SNPs falling into the same gene), the remaining markers identified 2,802 predicted genes annotated as *annotated protein* (2,440; 87.1%), sequence with *unknown function* (128; 4.6%) or *transcripts* (234; 8.3%).

### Identification of woolly poplar aphid resistance QTLs

**Fig 3:**
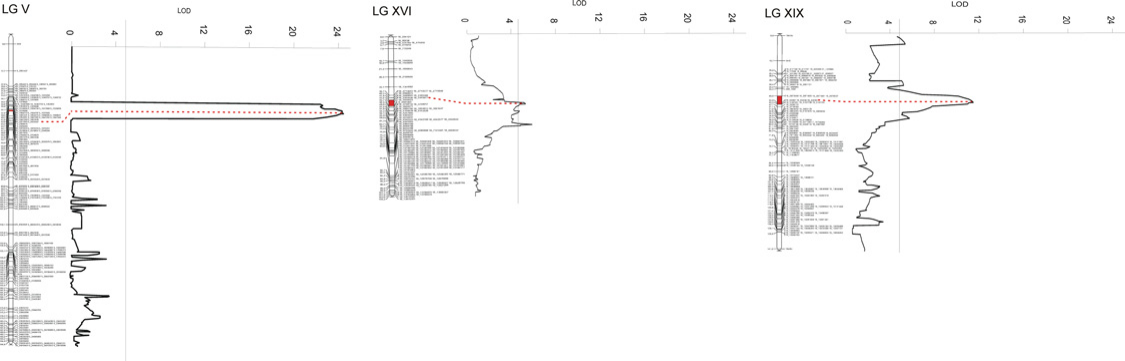
Chromosomal regions carrying the wpa resistance QTLs. The map position, the peak markers of each QTL and the LOD score are plotted. The horizontal line at 4.59 LOD value represents the LOD threshold determining the statistically significance of QTLs in the *P*. *deltoides* map.

Three significant QTL regions on LGV, LGXVI and LGXIX (named as wpa-5, wpa-16 and wpa-19, respectively) were identified on *P*. *deltoides* map. The percentages of phenotypic variance explained were 44.1% (wpa-5; LOD 23.6); 9.0% (wpa-16; LOD 6.6), and 17.4% (wpa-19; LOD 11.7). Epistasis analysis identified significant interaction (LOD 7.1) between wpa-5 and wpa-19 that contributed to the total of the phenotypic variation (65.8%) explained by the model (Fig 3 and Table 6). The QTL analysis performed in *P*. *nigra* parental line did not reveal any significant LOD value.

**Table 6:** Main features of the QTL regions for wpa resistance.

QTLs	LG	LOD	P value (Χ^2^)	Marker Interval (cM)	QTL peak position (cM)	QTL peak markers	Variance explained (%) ^b^
**wpa-5**	LG V	23.6	0.001***	5_1975251–5_ 2578834 (4.68)	43.7	5_2426240	44.1
**wpa-16**	LG XVI	6.6	0.1	16_2980973–16_3749017 (9.35)	43.4	16_3345538, 16_3345877	9.0
**wpa-19**	LG XIX	11.7	0.001***	19_2071803–19_3238172 (7.12)	44.8	78_83250, 78_83287, 78_83295	17.4
**Epistasis**	** **	** **					** **
wpa-5: wpa-19 **	* *	7.1	0.01**				9.7
Model		30.1	0				65.8

^b^ Percentage of variance explained at the peak of QTL

Signif. codes

‘***’ 0.001

‘**’ 0.01

‘*’ 0.05

### Putative candidate resistance genes in QTL regions

In plants, aphid resistance is mediated by several resistance mechanisms e.g. changes in the cuticle and metabolic rearrangements [31] and resistance (R)-genes encoding NBS-LRR proteins [32]. A search of putative candidate genes for wpa resistance functions was tentatively conducted by annotating the *P*. *trichocarpa* gene functions included in the confidence interval of the QTL regions (S2 Table). The wpa-5 locus resides on the chromosome 5 in the LOD-1 confidence interval between 1,975,251 bp and 2,578,834 bp of the *P*. *trichocarpa* reference genome, with the peak marker localized at 2,426,240 bp.

In this genomic interval, 82 expressed sequences were included, 75 of them were annotated and a large number were involved in disease resistance pathway (S2 Table). The wpa-5 peak marker (5_2426240) was mapped within a gene annotated as a NPK1-related protein kinase 1 (Locus POPTR_0005s03640; S2 Table), a class of genes recognized as involved in responses to biotic and abiotic stresses [33].

The wpa-16 locus was localized between 2,980,973 bp and 3,749,017 bp (chromosome 16) of the *P*. *trichocarpa* reference genome, within an interval where 100 putative genes were localized and 84 reported an annotation (S2 Table). Several genes coding for proteins potentially involved in poplar-aphid interaction were predicted for this genomic interval. They included five genes annotated as LRR protein kinase family and three zinc finger family proteins. The two co-segregating markers representing the wpa-16 peak (16_3345877, 16_3345538) were positioned at 3,345,877bp and 3,345,538 bp, respectively and both mapped on a gene annotated as “CCCH-type zinc finger protein with ARM repeat domain” (Locus ID: POPTR_0016s05410.1; S2 Table), which belongs to a class of genes involved in the regulation of development, growth and abiotic stress responses [34]. CCCH-type proteins are also involved in plant-pathogen interaction in rice, where it confers resistance to *Xanthomonas oryzae* [35] and in tobacco (*Nicotiana tabacum*) for *Rhizoctonia solani* resistance [36].

The region including the wpa-19 locus, corresponded to 7.12 cM in the *P*. *deltoides* map. In the *P*. *trichocarpa* genome this region was localized between 2,071,803 bp and 3,238,172 bp (chromosome 19). Within this genomic interval, a total of 146 expressed sequences were localized, 99 of them were annotated and a large number were identified as involved in disease resistance pathway (S2 Table). The three co-segregating markers (78_83250, 78_83287, 78_83295) representing the wpa-19 QTL peak were mapped in a gene annotated as Phospholipase A2 (Locus POPTR_0019s03100; S2 Table).

## Discussion

### Poplar linkage maps

An F1 population (131 individuals) derived from a *Populus deltoides* x *Populus nigra* cross, was genotyped with a total of 5,667 markers, 30 of them common in the two maps and 5,637 localized only in *P*. *nigra* or in *P*. *deltoides* map. 5,606 mapped markers possessed known physical position on *P*. *trichocarpa* sequenced genome. To our knowledge, these are the most saturated linkage maps developed for the genus *Populus*.

The number of markers mapped in the two poplar species was basically the same, but *P*. *nigra* map length (3,004.9 cM) was longer compared to *P*. *deltoides* map (2,745.2 cM). The *P*. *deltoides* map length reported in this work, was in agreement to previous studies, where the authors reported a range between 2,400 and 2,800 cM [2, 5]. A higher recombination frequency in the *P*. *nigra* genome could be causal of its larger map size.

The average density of framework markers in the *P*. *nigra* and *P*. *deltoides* maps developed in this work was almost the same (2.4 cM/locus in *P*. *nigra* and 2.3 cM/locus for *P*. *deltoides*) and allowed an unprecedented level of mean marker density compared to previously available maps where it was ranging from 5.2 cM/locus [5] to 6.9 cM/locus [6].

The bioinformatics analysis performed on 5,606 SNPs against *P*. *trichocarp*a cDNA v.2, revealed that almost all SNPs (99,75%) were localized into predicted genes. A relevant aspect of the two linkage maps developed in this work is that almost all markers can be used for a large-scale candidate gene identification. In addition, considering that *P*. *trichocarpa*, *P*. *nigra* and *P*. *deltoides* are the result of different evolutionary processes, the availability of more than 5,000 functional markers could be a resource to investigate evolutionary relationships of coding sequences among three poplar species cultivated around the world. Besides wpa resistance, *P*. *nigra* and *P*. *deltoides* are contrasting for other economical relevant traits such as the tolerance to *Marssonina* leaf spot (*Marssonina brunnea*) and to poplar leaf rust (*Melampsora larici-populina*), different plant habitus, different leaf form/dimension and different capability of roots development [10, 4]. All these traits are segregating in the developed *P* ×*canadensis* population and the obtained map can therefore be used to investigate the genetic bases of several other agronomically relevant poplar traits.

### Segregation distortion

Segregation distortion is common in mapping studies of forest trees and it has been documented in several *Populus* mapping works [5,37]. Distorted markers are not necessarily removed, as they may be linked to genes or traits of interest [4–5,37]. For this reason in our mapping studies markers distorted were included in the analysis.

Segregation distortion in hybrid pedigrees is frequently and commonly attributed to factors such as pollen-pistil incompatibilities [38], gametic competition [39], negative epistatic interactions among alleles [40–41], presence of ‘segregation distorter’ loci that result in the destruction of alternate gametes (meiotic drive [42]), or positive selection for the introgressing alleles [43–44]. At the base of the *P*. *deltoides* alleles competition, could exist an evolutionistic explanation, as hypothesize by Yin *et al*.[5]. The authors attributed the possibility of the allele selection to a variety of potential selective factors. In their *P*. *trichocarpa* x *P*. *deltoides* population several traits such as photoperiod sensitivity, frost tolerance and rooting ability were contributed by *P*. *trichocarpa*, since these traits are important for the survival of progeny, the segregation of markers associated with them could result in a distorted segregation.

In our work the number of distorted markers was almost the same between the two parents (264 in *P*. *deltoides* and 254 in *P*. *nigra*), but their distribution on the chromosomes of two parents varied in terms of block size, number and chromosomal regions. In the *P*. *nigra* map, five LGs (LG IV, LG VIII, LG XVI, LG XVII and LG XVIII) were free of segregation distortion, while *P*. *deltoides* map was characterized by only four segregation distortion free LGs (LGs VIII, XII and XIX). Four LGs in *P*. *deltoides* reported distorted segregation for more than >20% of the markers (LG VI, LG X, LGXVII and LG XVIII), while in *P*. *nigra* map only two LGs showed regions with high fraction of distorted markers (LG I-2, LG III), highlighting that the two maps did not share LGs bearing highly distorted markers. An interesting aspect of this investigation was the extensive occurrence of segregation distortion in favor of *P*. *deltoides* alleles in LG XVII of *P*. *deltoides* map and in LG III of *P*. *nigra* map. The same findings about unidirectional distortion of marker alleles were observed in a *P*. *trichocarpa x P*. *deltoides* cross for the *P*. *deltoides* alleles [5].

### QTL analysis and wpa resistance

No genetic information was currently available about plant response and resistance QTL against wpa, one of the most damaging pest in poplar. Some information was available about the plant-aphid interaction such as type of poplar resistance (antibiosis or antixenosis) by assessing aphid settlement, physiology and stylet penetration behavior [9], but no candidate genes or chromosomal regions were, to our knowledge, described. Aphid colonization was studied also in other species and several defense pathways are known to confer resistance, including the involvement of secondary metabolites such as quercetin or isorhamnetin in cowpea [45], drastic reductions in phloem sap ingestion by aphids (*Myzus persicae*) in peach trees [46] and elicitation of defense reactions based on salicylic acid (SA), jasmonic acid (JA), ethylene and brassinosteroids signaling pathways in tomato-aphids interaction [47].

Resistance to aphids is frequently controlled by major dominant or semi-dominant genetic loci homologues to the R-genes conditioning pathogen resistance [48]. In apple, three resistance genes (*Rvi 16*, *Er4*, *Pl-m*), associated to woolly apple aphid (*Eriosoma lanigerum*) have been identified [49]; in melon a dominant locus, *Vat*, coding for coiled-coil–nucleotide-binding-site–leucine-rich repeat (CC-NBS-LRR) protein type confers a high level of resistance to cotton aphid (*Aphis gossypii*) and virus infestation [50]; in soybean one NB-LRR class gene (*Raso 2*) confers both antixenosis and antibiosis resistance responses to foxglove aphid (*Aulacorthum solani*) [51]. Less is known about aphid resistance in forest trees and no genetic information about Poplar–wpa interaction or genetic factors controlling wpa resistance is currently available. In a recent work, Dardeau *et al*. [11] tested in three *P* ×*canadensis* hybrids the wpa response at anatomical and biochemical levels through histological and histochemical approaches. The authors reported a similar response to mechanical wounding (such as intense lignins, tannins and flavanol deposition) between resistant and susceptible genotypes, while they discovered a different response to the probing by wpa among genotypes. Hyperplasia and cell hypertrophia, associated to galls formation were found in susceptible genotypes, affecting sap conduction and the tree survival.

Even if the F1 mapping population was not large, three QTL regions (wpa-5, wpa-16 and wpa-19 localized on LG V, LG XVI and LG XIX, respectively), explaining a considerable fraction (65.8%) of the phenotypic variability between *P*. *deltoides* (resistant parental line) and *P*. *nigra* (susceptible parental line) were identified. The marker representing the wpa-5 QTL peak, the major QTL explaining 44.1% of variability, was mapped on LGV in a gene predicted to encode a NPK1-related protein kinase 1. In Arabidopsis, the expression of a kinase-negative mutant of NPK1 resulted in the generation of multinucleate cells with incomplete cell plates formation during cytokinesis. Phragmoplasts were formed, but their expansion toward the cell cortex was blocked. In addition, the authors discovered that NPK1 played a function also in the process of the proper nuclear separation, suggesting that NPK1 might regulate both cytokinesis, both the proper nuclear separation [52]. Since wpa induced pseudogalls, an abnormal cell proliferation, in susceptible genotypes [11], the NPK1-related protein kinase kinase gene product could represent a possible function encoded by the wpa-5 QTL. Moreover, Kovtun *et al*.[33] reported that the overexpression of the kinase domain of NPK1 or its Arabidopsis ortholog ANP1, resulted in suppression of auxin and in activation of oxidative stress signaling, while in the expression of NPK1 in maize induced a set of similar stress genes, such as Glutathione-S-transferase (GST), Heat Shock Proteins (HSPs), and pathogen related 1 protein (PR1), indicating that this protein plays an important role in the oxidative signaling cascade [53]. All these findings suggested that NPK1 family might be multifunctional in plant cells and certainly involved in stress response mechanisms.

In the wpa-16 QTL region in *P*. *trichocarpa*, genes encoding for proteins involved in pathogen interaction included Leucine Rich Repeat (LRR) protein kinase family and zinc finger family proteins. The two marker peaks were mapped on a gene annotated as “CCCH-type zinc finger protein with ARM repeat domain”, functioning in the regulation of development, growth, or abiotic stress responses [34]. CCCH-type proteins are involved in plant-pathogen interactions in rice, conferring resistance to *Xanthomonas oryzae* [35] and in tobacco (*Nicotiana tabacum*) where enhanced resistance to *Rhizoctonia solani* [36].

In the wpa-19 region, a cluster of 14 genes coding for TIR-NB-LRR disease resistance proteins genes was identified. Several works revealed the presence on chromosome 19 of disease resistance genes and resistant phenotypes because many of the poplar NB-LRR genes (more than 70,) are gathered into a supercluster localized on that chromosome [54]. The largest NB–LRR gene supercluster on *Populus* chromosome 19 co-localized with the resistance loci MER, R1, and RUS,. All the genes cited are either inherited from *P*. *deltoides* or *P*. *trichocarpa* conferring qualitative or quantitative resistance to *Melampsora larici-populina* in *P*. *deltoides* and hybrid poplar, derived from the mating [55–57]. The three peak markers of wpa-19 were localized in a sequence annotated as “Phospholipase A2” (PLA2), a class of proteins associated to signal transduction pathways and implicated in plant-pathogen interactions [58–60].

PLA2 accumulation is dependent on ethylene and jasmonic acid signaling produced during responses to pest attack and regulating sets of defense genes [61].

A number of genes were therefore identified under the genomic interval of the QTL regions whose encoded functions could be compatible with a role in wpa resistance. It should however be considered that the species used in this study to investigate wpa resistance were *P*. *nigra* and *P*. *deltoides* while the reference genome sequence in which these genes were identified is *P*. *trichocarpa*. The gene content of the identified intervals could therefore be substantially different across these distantly-related species. However, the identification of putative candidates utilizing a reference genome can provide valuable contribution in terms of additional molecular markers that can be added to a high resolution genetic map and, following verification of maintained positional relationships after fine mapping, can provide sequences that can be exploited in the analysis of allelic diversity among resistant and susceptible genotypes.

## Conclusions

A successful application of the GBS approach allowed the development of two linkage maps in *P*. *deltoides* and *P*. *nigra* based mostly on gene-based SNPs. The two maps were subsequently employed in QTL mapping of wpa resistance, leading to the identification of resistance QTLs on LG V, XVI and XIX. The *P*. *trichocarpa* reference genome sequence was exploited for the identification of candidate genes underlying the resistance QTLs and candidate genes encoding proteins implicated in defense pathways were identified. Additional steps of high-resolution mapping will further improve the identification of candidate genes for the resistance QTLs. Finally, the SNPs markers associated to *P*. *deltoides* wpa resistance QTLs represent valuable tools for marker-assisted introgression of the resistant loci into susceptible poplar genotypes.

## Supporting Information

S1 Fig Genetic linkage maps of *P*. *deltoides and P*. *nigra*. The grey line on the left of chromosomes represents the region where segregated distorted markers mapped.
(RAR)

S1 Table List of the annotated genes, referred to *P*. *trichocarpa* genome, identified by the mapped SNPs. (XLSX)

S2 Table List of the candidate genes, referred to *P*. *trichocarpa* genome, included in the confidence interval of the three QTL regions. (XLSX)
